# Development of IVF Porcine Embryos in Microwell Culture System

**DOI:** 10.3390/ani15172528

**Published:** 2025-08-28

**Authors:** Kayode Balogun, Zoltan Machaty

**Affiliations:** Department of Animal Sciences, Purdue University, 270 S. Russell Street, West Lafayette, IN 47907, USA; kbalogu@purdue.edu

**Keywords:** embryo, in vitro fertilization, oocyte, pig, well-of-the-well system

## Abstract

We investigated the development of IVF porcine embryos incubated in microwells of Well-of-the-Well (WOW) culture dishes. In Experiment 1, 500 embryos were cultured under five different culture conditions: 10 individual embryos in microwells covered by 50 μL PZM-3 medium (50 μL WOW); 10 embryos together in a well of 50 μL PZM-3 (50 μL Well); 10 embryos in microwells covered by 20 μL PZM-3 (20 μL WOW); 10 embryos in a well of 20 μL PZM-3 (20 μL Well); and 10 embryos in a 20 μL drop of PZM-3 in traditional dishes (control). In Experiment 2, the best-performing treatment group from Experiment 1 (50 μL WOW) was further compared to control using a total of 350 embryos. There was no difference in the percentage of cleaved embryos among the groups in both experiments. In Experiment 1, the percentage of blastocysts from 50 μL WOW (42.00 ± 6.29%) was higher (*p* = 0.001) than in 50 μL Well (29.00 ± 4.58%), 20 μL WOW (13.00 ± 2.13%), 20 μL Well (20.00 ± 2.98%) and control (25.00 ± 5.22%). Similarly, in Experiment 2, blastocyst formation and nuclei number in the blastocysts from 50 μL WOW (37.86 ± 3.95% and 38.97 ± 1.80) were higher (*p* = 0.040 and *p* = 0.017) than in the control (28.10 ± 2.64% and 33.21 ± 1.56). The results indicate that individual culture in microwells improves the development of IVF pig embryos.

## Simple Summary

The efficiency of producing porcine embryos in a laboratory by in vitro fertilization (IVF) is notoriously low. Our understanding of the relevant factors contributing to this low efficacy is limited, in part, by the difficulty of tracking the development of individual embryos. Porcine embryos are known to develop better when cultured in groups, and there is a need for a culture system that enables the individual culture of embryos. We investigated the optimal culture conditions for IVF pig embryos incubated in Well-of-the-Well (WOW) culture dishes. Our findings demonstrate, for the first time, that porcine embryos produced by in vitro fertilization can be individually cultured in microwells without compromising their developmental competence, allowing for detailed individual assessments and evaluations. In addition to this, we identified an ideal volume of culture medium necessary to maximize both the quantity and quality of the resulting blastocysts. These findings are significant in advancing both the efficiency of porcine embryo production in vitro and the capability to track individual embryo development, which may be valuable for a variety of experimental and applied research purposes.

## 1. Introduction

Producing pig embryos in a laboratory offers advantages in both agriculture and biomedicine. Their use in agriculture may help to maximize genetic improvement and curb the transmission of diseases, whereas in medicine, pigs—because of their similarity to humans in size, physiology, and genetics—are considered ideal as models of human genetic diseases [1,2,3]. Unfortunately, the success rate of assisted reproductive technologies is relatively low [4,5]. A major reason for this is that most technologies involve the handling of embryos under suboptimal conditions in the laboratory. For the advancement of numerous assisted reproductive technologies, a better understanding of the factors that affect the in vitro development of porcine embryos is highly desirable.

In the laboratory, preimplantation mammalian embryos are usually cultured in groups in order to achieve better developmental rates. This is because, as various studies have shown, such embryos communicate with each other by producing and exchanging embryonic factors that have paracrine and autocrine actions. These factors assist in the developmental process and support the viability of neighboring embryos [6,7,8]. The beneficial effects of the embryo secretome during early development and the paracrine action of numerous compounds produced by the embryos have been highlighted in several studies [9,10]. Lower-quality embryos, on the other hand, produce detrimental factors that have a negative effect on embryo development. Despite the possible harmful factors, porcine embryos—like most other species—are usually incubated in groups, preferably in groups of at least 10, and their development, when cultured individually, seems to be impaired due to the absence of a substantial amount of embryo-derived beneficial molecules [11].

Although individual culture of embryos has its potential disadvantages, it may be desirable from specific standpoints, a primary advantage being the opportunity to monitor the fate and developmental status of each embryo during the culture period. Although the embryos incubated separately lack the support of other embryos and develop poorly, culturing them in microwells seems to circumvent this problem. The Well-of-the-Well (WOW) culture dish has microwells that can hold individual embryos, while they are covered by a common drop of culture medium [12]. Thus, the dish offers the possibility of culturing several embryos at the same time in individual wells, and their development can be monitored during the entire culture period. Individual cultures have been investigated in the case of mouse, bovine, and human embryos produced by in vitro fertilization, and also in porcine embryos generated by parthenogenetic activation or intracytoplasmic sperm injection [8,10,12,13]. Because of the importance of generating high-quality pig embryos for agricultural and biomedical research, and because specific parameters of the microwell culture have been shown to impact preimplantation embryo development [14], the present study was designed to determine ideal conditions for the individual culture of IVF pig embryos in microwells of the WOW culture dish.

## 2. Materials and Methods

### 2.1. In Vitro Oocyte Maturation

Ovaries of prepubertal gilts used for this study were sourced from a local slaughterhouse (Indiana Packers Corporation; Delphi, IN, USA) and transported to the laboratory in physiological saline. The content of medium-sized follicles (3–6 mm in diameter) was aspirated using an 18-gauge hypodermic needle attached to a 10-mL syringe. Cumulus-oocyte complexes (COCs) were selected and washed twice in HEPES-buffered Tyrode’s lactate (TL-HEPES) medium in a Petri dish using a stereomicroscope (Nikon SMZ645, Nikon Corporation, Tokyo, Japan). COCs with oocytes having evenly granulated cytoplasm and an intact cumulus investment were placed into the maturation medium (50 COCs into 500 μL medium). The maturation medium consisted of TCM-199 (Invitrogen, Grand Island, NY, USA) supplemented with 3.05 mM glucose, 0.91 mM sodium pyruvate, 0.57 mM cysteine, 10 ng/mL epidermal growth factor (EGF), 0.5 IU/mL ovine luteinizing hormone (LH), 0.5 IU/mL porcine follicle stimulating hormone (FSH), and 0.1% polyvinyl alcohol (PVA), 75 mg/mL penicillin, and 50 mg/mL streptomycin. COCs were allowed to mature for 44 h under regulated conditions of 39 °C in an atmosphere of 5% CO_2_ in air with 100% humidity [15].

### 2.2. In Vitro Fertilization (IVF)

At the end of the maturation period, COCs were vortexed in TL-HEPES containing 1 mg/mL hyaluronidase for 5 min to remove the cumulus cells from the zonae pellucidae. Only oocytes with intact plasma membrane, evenly dark cytoplasm, and an extruded first polar body were selected and used in the experiments. Denuded oocytes were rinsed in a fertilization medium (modified Tris-buffered medium; mTBM) which contains 113.1 mM NaCl, 3 mM KCl, 7.5 mM CaCl_2_ × 2H_2_O, 20 mM Tris (crystallized free base), 11 mM glucose, 5 mM sodium pyruvate, 0.1% bovine serum albumin (BSA), and 1mM caffeine. Groups of 30 oocytes were transferred into 50 μL droplets of the medium covered with mineral oil. Frozen-thawed boar semen was used for IVF. Straws of frozen semen were thawed at 50 °C for 20 s and the semen was then emptied into 10 mL of 39 °C Dulbecco’s phosphate-buffered saline (DPBS) inside of a 15 mL centrifuge tube. The suspension was rinsed three times by centrifugation at 900× *g* for 4 min in fresh DPBS. The final sperm pellet was mixed in mTBM to a concentration of 1 × 10^6^ sperm/mL. Fifty µL of the sperm suspension was added to the 50 μL mTBM droplet containing the 30 selected oocytes, so the final concentration in 100 µL fertilization drops was 0.5 × 10^6^ sperm/mL. The gametes were then allowed to co-incubate for a period of 5 h [15].

### 2.3. Embryo Culture

**Figure 1:**
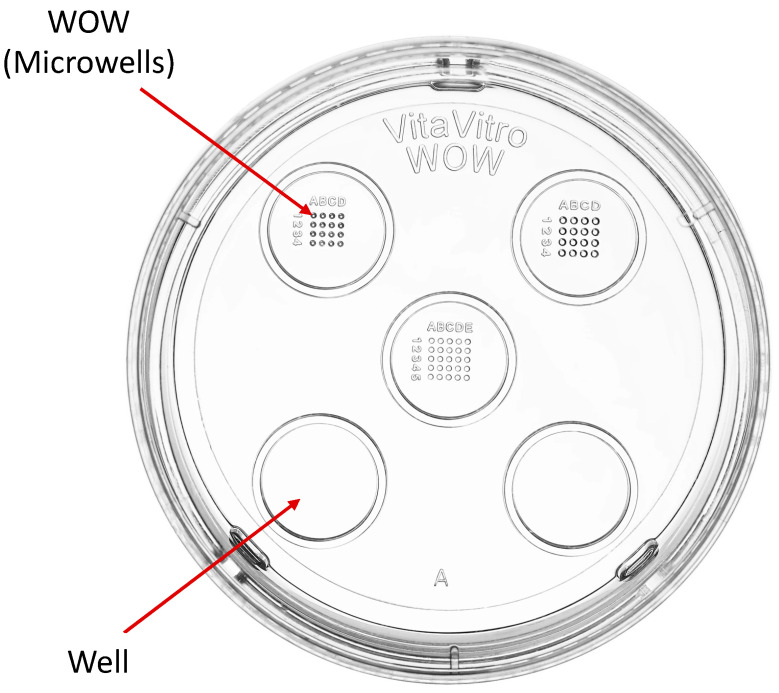
Well-of-the-Well culture dish [16].

The Well-of-the-Well embryo culture dish used for this study was sourced from VitaVitro Biotech (Shenzhen, China). The dish comprises wells and microwells inside the wells, as shown in Figure 1. The approximate width and depth of each round-bottom conical shape impression of the WOW used (upper left compartment) are 250 and 200 μm, respectively. The dimensions of the whole dish are 1 cm in height and 35 mm in diameter.

In Experiment 1, the presumed zygotes were rinsed three times in the culture medium (Porcine Zygote Medium 3; PZM-3) [17] and transferred into five different groups of PZM-3 drops (10 embryos per drop). These groups included (1) embryos cultured individually in microwells of the WOW dish covered by 50 μL of PZM-3 (50 μL WOW); (2) embryos cultured together in 50 μL of PZM-3 in a well of the same dish (50 μL Well); (3) embryos cultured individually in microwells covered by 20 μL of PZM-3 medium (20 μL WOW); (4) embryos cultured together in 20 μL of PZM-3 medium in a well of the same dish (20 μL Well); and (5) embryos cultured together in a 20 μL drop of PZM-3 medium in a conventional small Petri dish (control). Medium volumes were held constant across experiments. The drops and the other treatment groups were covered with mineral oil and incubated for 7 days under the same conditions used for oocyte maturation, which has been previously validated to support porcine blastocyst development in vitro [18]. Each replicate represents a separate IVF experiment using different oocyte collections and independently thawed semen aliquots from the same boar. A total of 500 embryos were used in this experiment. The number of cleaved embryos in each group was determined 24 h after the beginning of in vitro fertilization, and at the end of the culture period, the number of blastocysts was also recorded. Only embryos that exhibited a well-defined, expanded blastocoel cavity and observable inner cell masses were classified as blastocysts across experiments.

In Experiment 2, the presumed zygotes were rinsed three times in PZM-3 culture medium and transferred into two groups of PZM-3 drops (10 embryos per drop). A total of 350 embryos were allocated into two groups during the course of this experiment. These groups included the best-performing treatment in Experiment 1 (50 μL WOW) vs. 20 μL drop (control). The culture medium drops were covered with mineral oil and incubated for 7 days under the same conditions as described in Experiment 1. Each replicate represents a separate IVF experiment using different oocyte collections and independently thawed semen aliquots from the same boar. The number of cleaved embryos was determined 24 h after the beginning of in vitro fertilization, and at the end of the culture period, the number of embryos that reached the blastocyst stage was recorded. The number of nuclei in the blastocysts was also determined.

### 2.4. Nuclear Number of Blastocysts

After 7 days of culture, embryos that reached the blastocyst stage were selected and the nuclear number of each embryo was determined. For this purpose, the blastocysts were first placed in a drop of 500 μL DPBS containing 1 μg/mL of the nuclear stain Hoechst 33,342 for 5 min. The stained blastocysts were subsequently rinsed in another 500 μL drop of DPBS briefly before they were mounted on microscope slides under posted coverslips. The number of nuclei was determined using epifluorescence microscopy. To minimize the limitations of this method, we carefully excluded extrazygotic sperm from the counts based on nuclear size, shape, and location relative to embryonic nuclei.

### 2.5. Statistical Analysis

In Experiment 1, embryo development data were analyzed using the Mixed Procedure of the Statistical Analysis System (SAS 3.82; Enterprise Edition). In Experiment 2, *t*-test of SAS was used to analyze embryo development and nuclei count data. Significant differences among means ± SEM were identified at *p* ≤ 0.05.

## 3. Results

**Figure 2:**
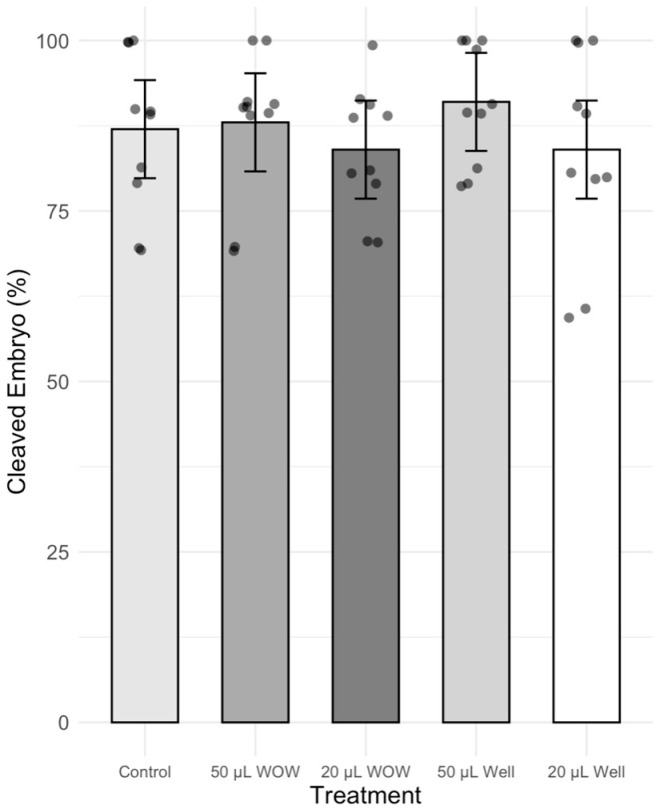
Bar graph showing the means ± SEM with scatter plots of replicates of percentage cleaved porcine embryos during culture under different conditions. Control—20 μL drop of PZM-3 in conventional culture dish; 50 μL WOW—50 μL of PZM-3 in Well-of-the-Well dishes; 20 μL WOW—20 μL of PZM-3 in Well-of-the-Well dishes; 50 μL Well—50 μL of PZM-3 in well; and 20 μL Well—20 μL of PZM-3 in well. *p* = 0.608. Ten replicates were performed with 100 embryos per experimental treatment and 500 embryos in total.

**Figure 3:**
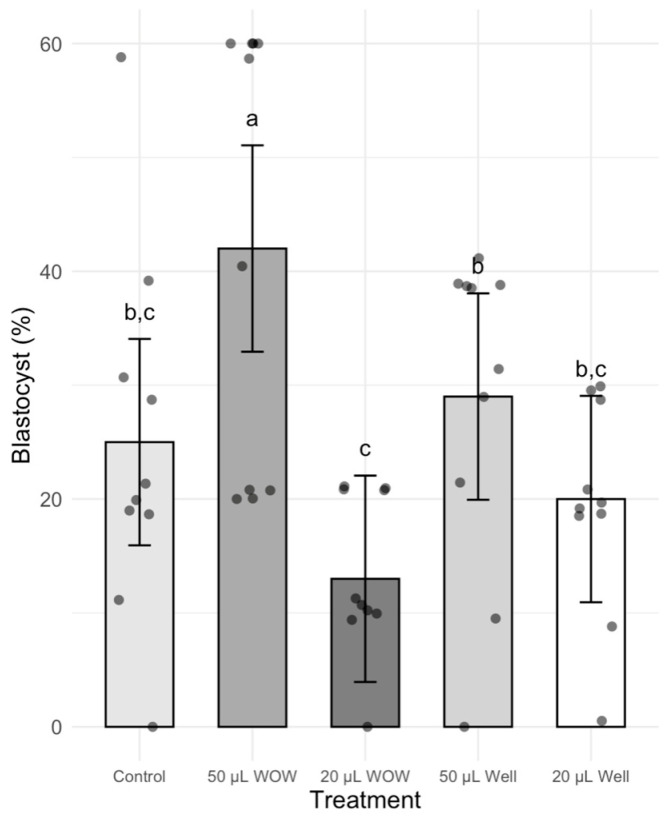
Bar graph showing the means ± SEM with scatter plots of replicates of percentage blastocysts following incubation of the embryos under different conditions. Different superscript letters (a, b, and c) indicate significant differences (*p* = <0.001). Control—20 μL drop of PZM-3 in conventional culture dish; 50 μL WOW—50 μL of PZM-3 in Well-of-the-Well dishes; 20 μL WOW—20 μL of PZM-3 in Well-of-the-Well dishes; 50 μL Well—50 μL of PZM-3 in well; and 20 μL Well—20 μL of PZM-3 in well. *p* = 0.608. Ten replicates were performed with 100 embryos per experimental treatment and 500 embryos in total.

**Figure 4:**
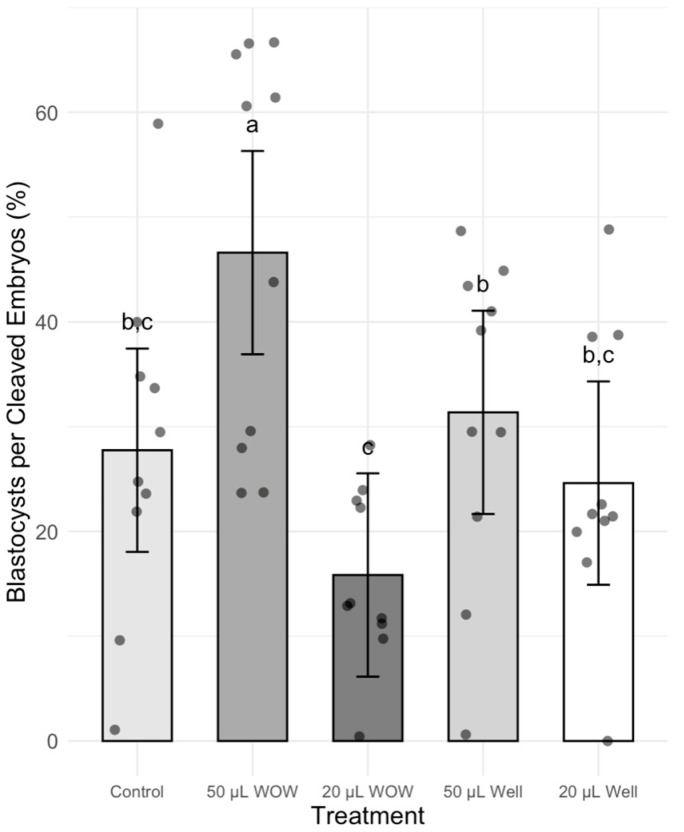
Bar graph showing the means ± SEM with scatter plots of replicates of percentage blastocysts per cleaved embryos following incubation of the embryos under different conditions. Different superscript letters (a, b, and c) indicate significant differences (*p* = 0.001). Control—20 μL drop of PZM-3 in conventional culture dish; 50 μL WOW—50 μL of PZM-3 in Well-of-the-Well dishes; 20 μL WOW—20 μL of PZM-3 in Well-of-the-Well dishes; 50 μL Well—50 μL of PZM-3 in well; and 20 μL Well—20 μL of PZM-3 in well. *p* = 0.608. Ten replicates were performed with 100 embryos per experimental treatment and 500 embryos in total.

**Figure 5:**
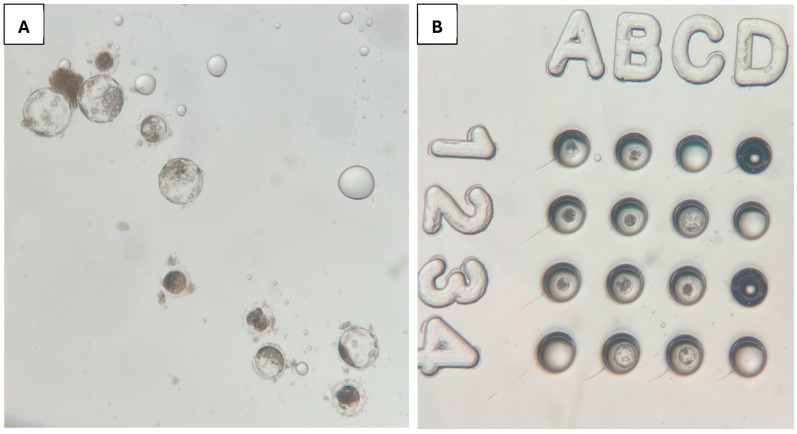
Porcine embryos after 7 days of culture in a conventional embryo culture Petri dish (**A**) and a Well-of-the-Well embryo culture dish (**B**).

There was no significant difference in the percentage of cleaved embryos among treatment groups, which ranged between 84 and 91% (Figure 2). However, the percentage of blastocysts in the 50 μL WOW group was significantly higher (42.00 ± 6.29%; *p* < 0.001) than in the 50 μL Well (29.00 ± 4.58%), 20 μL WOW (13.00 ± 2.13%), 20 μL Well (20.00 ± 2.98%) and control (25.00 ± 5.22%) groups (Figure 3). Similarly, the percentage of blastocysts per cleaved embryos in the 50 μL WOW group was significantly higher (46.60 ± 6.16%; *p* = 0.001) than in the 50 μL Well (31.36 ± 5.02%), 20 μL WOW (15.84 ± 2.71%), 20 μL Well (24.61 ± 4.37%) and control (27.75 ± 5.15%) groups (Figure 4). Typical embryos formed in the various culture systems after 7 days of culture are shown in Figure 5.

**Figure 6:**
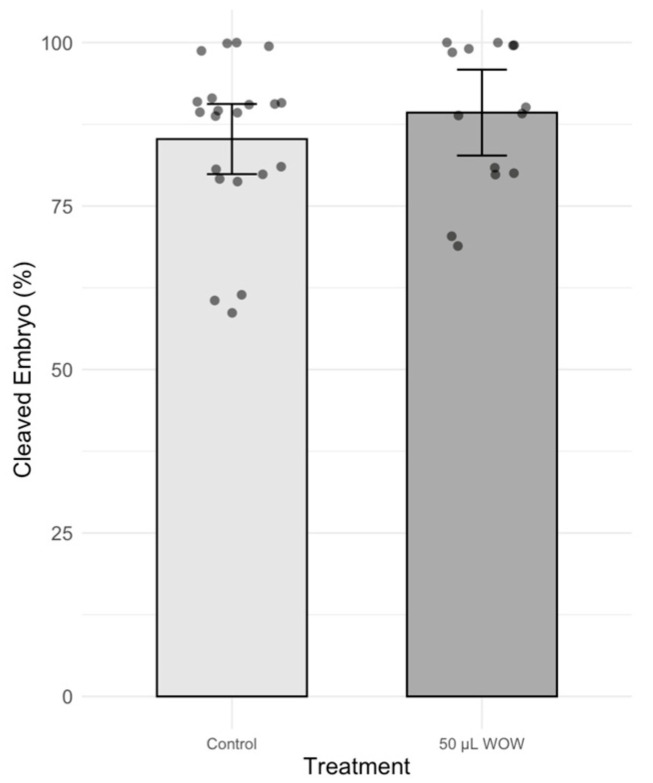
Bar graph showing the means ± SEM with scatter plots of replicates of percentage cleaved porcine embryos during culture under different conditions. Control—20 μL drop of PZM-3 in conventional culture dish. 50 μL WOW—50 μL of PZM-3 in Well-of-the-Well dishes. *p* = 0.339. A total of 350 embryos were used in this experiment.

**Figure 7:**
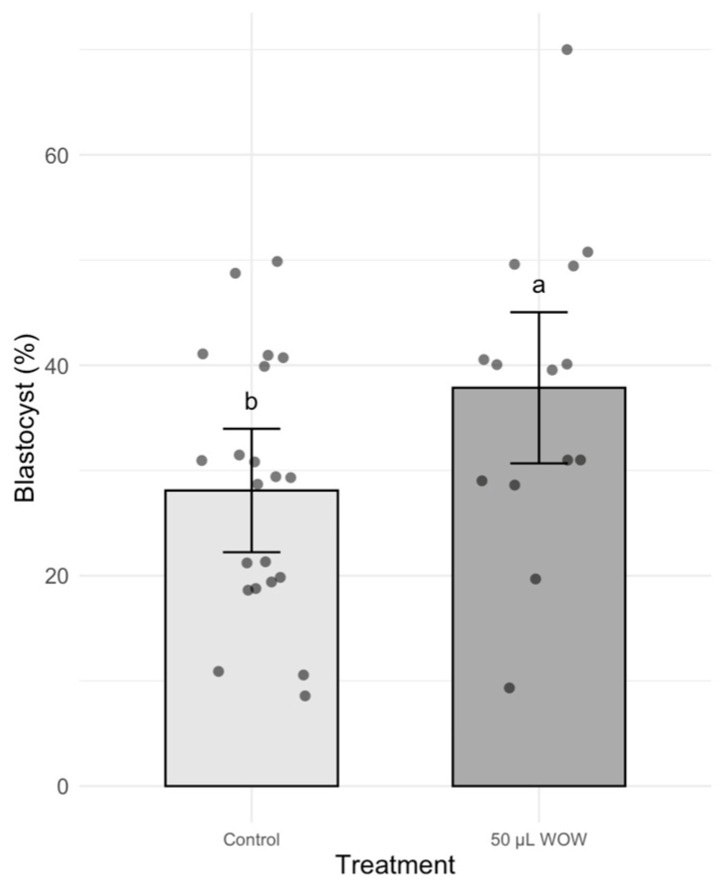
Bar graph showing the means ± SEM with scatter plots of replicates of percentage blastocysts following incubation of the embryos under different conditions. Different superscript letters (a and b) indicate significant differences (*p* = 0.040). Control—20 μL drop of PZM-3 in conventional culture dish. 50 μL WOW—50 μL of PZM-3 in Well-of-the-Well dishes. A total of 350 embryos were used in this experiment.

**Figure 8:**
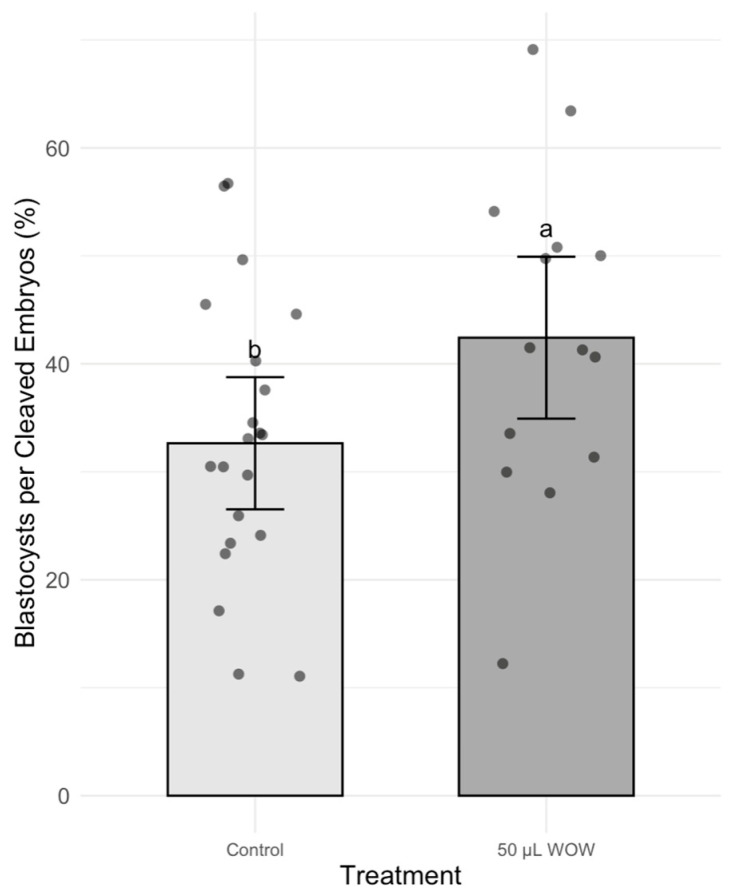
Bar graph showing the means ± SEM with scatter plots of replicates of percentage blastocysts per cleaved embryos following incubation of the embryos under different conditions. Different superscript letters (a and b) indicate significant differences (*p* = 0.048). Control—20 μL drop of PZM-3 in conventional culture dish. 50 μL WOW—50 μL of PZM-3 in Well-of-the-Well dishes. A total of 350 embryos were used in this experiment.

**Figure 9:**
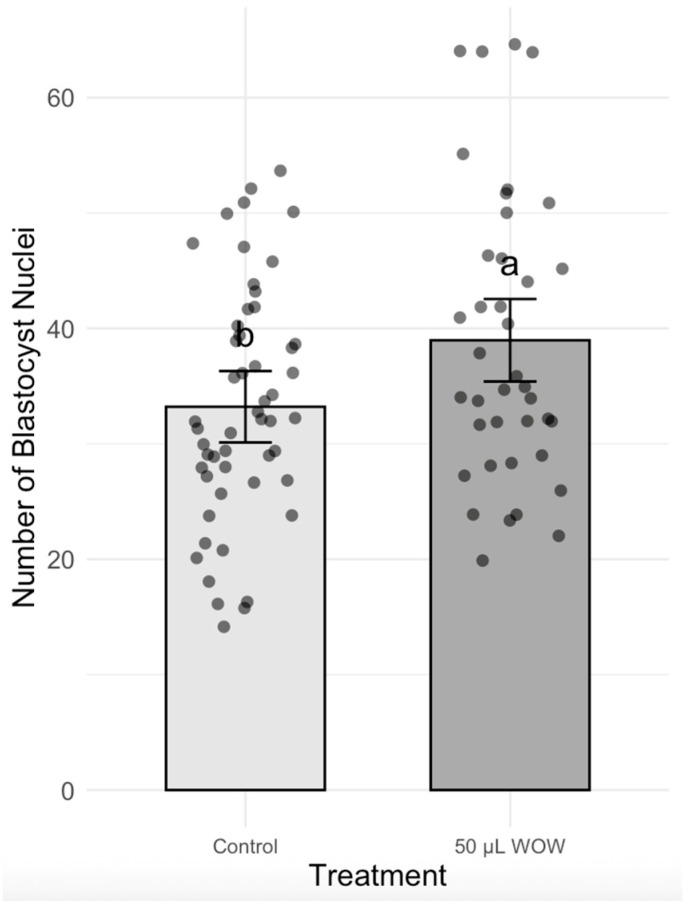
Bar graph showing the means ± SEM with scatter plots of the number of nuclei in blastocysts after culturing the embryos under different conditions. A total of 91 embryos were used in this experiment. Different superscript letters (a and b) indicate significant differences (*p* = 0.017).

**Figure 10:**
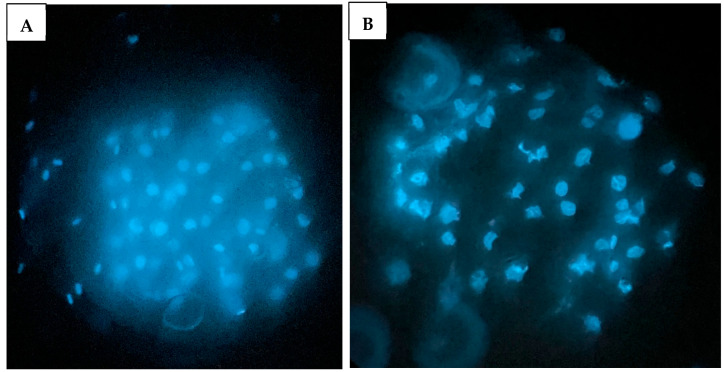
Porcine blastocysts stained with Hoechst 33,342 to determine the number of nuclei in the embryo. (**A**) Embryo from 50 μL WOW treatment group; (**B**) Embryo from control group. The smaller blue oblong structures represent sperm nuclei, while the larger, rounder blue structures indicate nuclei in the embryonic cells.

In Experiment 2, the treatment group that resulted in the highest blastocyst development in the previous experiment (50 μL WOW) was further compared to the control group. Among the 350 embryos used in this experiment, there was no difference in the percentage of cleaved embryos as shown in Figure 6 (control, 85.24 ± 2.73% vs. 50 μL WOW, 89.29 ± 3.05). However, the percentage of blastocysts formed in the 50 μL WOW group (37.86 ± 3.95%) was again significantly higher than in the control group (28.10 ± 2.64%; *p* = 0.040) (Figure 7). Additionally, the percentage of blastocysts per cleaved embryo in the 50 μL WOW group (42.42 ± 4.12%) was again significantly higher than in the control group (32.64 ± 2.76%; *p* = 0.048) (Figure 8). Finally, when we analyzed the average number of nuclei in the blastocysts formed under the different culture conditions, we found that the number of nuclei in the blastocysts of the 50 μL WOW group (38.97 ± 1.80) was significantly higher than in the control group (33.21 ± 1.56; *p* = 0.017) (Figure 9). Typical nuclear staining of the blastocysts is shown in Figure 10.

## 4. Discussion

The utilization of in vitro-produced porcine embryos for research and commercial pig production continues to be limited due to poor developmental rates, low pregnancy rates, and small liter size [19]. In order to better understand the factors that affect development, determining the timing and progression of specific developmental stages such as cleavage and blastulation is useful as these can be predictors of embryo viability. However, observing such developmental cornerstones in the case of individual embryos is challenging because embryos develop poorly when incubated separately. The culture of embryos individually resulted in inferior blastocyst formation and low cell number [20,21,22] and it is now generally accepted that the embryos show better development when cultured together as a group, when they are supported by embryotrophic autocrine and paracrine factors secreted by the embryos [12]. However, tracking the progression of development of individual embryos has practical importance because it facilitates the monitoring of specific biomarkers that may reveal the viability and developmental potential of the embryos. In this study, we investigated whether porcine embryos produced by in vitro fertilization can be cultured individually in microwells of the Well-of-the-Well culture dish without impediment in their development. We also wanted to find out the conditions that best support development to the blastocyst stage during individual culture.

It is well documented that group culture has benefits on the developmental processes of early embryos. Although their mechanism of action is not properly understood, autocrine (factors that are produced by the blastomeres) and paracrine factors (those that are produced by the trophectoderm or the inner cell mass and are involved in the interaction of these cells) are known to play a crucial role in modulating the development of mammalian embryos [23]. Their studies began a few decades ago when it was recognized that the embryos can condition their own medium: their in vitro development was better when they were incubated in small drops of culture medium instead of large drops [20,24]. Those that are best characterized include members of the transforming growth factor beta (TGF-β) family, the epidermal growth factor (EGF) family, the insulin and insulin-like growth factor (IGF) family, leukemia inhibitory factor (LIF), colony-stimulating factor 1 (CSF-1), tumor necrosis factor-α (TNF-α), interleukin-1 (IL-1), platelet-derived growth factor (PDGF), and platelet-activating factor (PAF) [23,25]. They exist naturally in the reproductive tract of the dam; either the factors, their receptors, or both, are expressed by the preimplantation embryo. It was proposed that the presence of these factors is responsible for the higher developmental rates of embryos observed in vivo as opposed to in vitro in both cattle and pigs [26,27]. In fact, the supplementation of the culture medium of mouse and bovine embryos with growth factors such as IGF-I, IGF-II, PAF [22], or PDGF [28] could compensate for the adverse effects of culture in large volumes and lead to an improvement in development. It is, however, imperative to distinguish between paracrine signaling, which are interactions between neighboring embryos through secreted factors [6,26], and intraembryonic signaling, such as communication between trophectoderm and inner cell mass inside an embryo [29]. Additionally, intracrine signaling, where signaling molecules act within the same cell that produces them, may also play a crucial role in regulating early embryonic development [30].

In addition, an increase in embryo density in the culture drop improved the percentage of mouse embryos undergoing compaction [31], cavitation [20,32], hatching [31], and implantation [21]. Similar findings were reported in cattle, where group culture had a positive effect on blastocyst formation, cell number, and the production of interferon-tau by the embryos [33,34,35]. The result of the present study suggests that these paracrine and autocrine factors that are essential for proper embryo development may be available not only for embryos developing in groups but also for those that are cultured individually in microwells, sharing the same drop of culture medium. In fact, we found that the best development was achieved in the 50 μL WOW group, where the blastocyst rate was the highest compared to any other treatment group, including the control. The better result of blastocyst formation suggests that the essential factors are perhaps localized closer to, and therefore can be utilized more easily by, the embryos inside the microwells. Blastocyst formation relative to cleaved embryos further confirmed the advantage of the 50 μL WOW system, highlighting its ability to support post-cleavage development independent of fertilization variability. It has been proposed that in the WOW system, detrimental metabolic products are diluted sufficiently, and autocrine/paracrine factors are accumulated appropriately so that the system supports superior embryo development [8,12]. This postulation may explain the higher blastocyst development in the 50 μL WOW group compared to the 50 μL Well group (which developed in the same volume of medium in wells without microwells), indicating better utilization of the available resources within the same culture drop volume.

Another important aspect regarding embryo culture is the volume of the culture medium. The optimal size of the droplet should minimize the accumulation of the toxic metabolites and, at the same time, maintain the advantageous autocrine and paracrine factors around the embryos. There is no total agreement on the optimal droplet size and what is related to this, the ideal embryo-medium ratio. Some studies reported that bovine embryo quality markedly improved when the embryos were cultured in reduced volumes of culture media, and increasing the size of the medium droplet had a negative effect on development [36,37]. Others found that the development of mouse embryos improved with decreasing embryo density in the culture drop [14]. It was hypothesized that the higher volume allows for more availability of nutrition and perhaps a less harmful environment from a build-up of toxic metabolites or reactive oxygen species (ROS) [38,39]. In a culture drop environment, the embryos release waste products in the form of metabolites. These metabolic waste products, particularly ammonium, increase toxicity in the culture medium drop [40,41,42]. In addition, low-quality embryos have been shown to release harmful factors, including kallikrein (KLKB1) and vitamin D-binding protein, that affect embryo development negatively [10]. In the present study, uncleaved embryos were not separated during culture, and all embryos were maintained in their assigned groups throughout the experiment. However, we observed relatively high and consistent cleavage rates across treatment groups (ranging from 84.00 ± 3.06% to 91.00 ± 2.77%), indicating that the proportion of low-quality embryos per group was relatively low and unlikely to have substantially influenced the development of neighboring embryos and the overall developmental outcomes. In practice, a culture drop volume of 20 μL is frequently used because it supplies sufficient nutrient concentration to support embryo metabolic needs while preventing the harmful accumulation of waste material. In our experiments, the idea that a higher volume of culture medium in the WOW system, at 50 μL, can improve blastocyst development materialized in the microwell environment (42.00 ± 6.29% blastocyst formation in the 50 μL WOW vs. 13.00 ± 2.13% in the 20 μL WOW system). This result is similar to what others found in the case of mouse embryos, i.e., a 50 μL droplet of culture medium was superior in supporting development compared to 6.25, 12.5, and 25 μL droplets, each containing 9 embryos [14]. Furthermore, the observation that 20 μL of PZM-3 spreads unevenly and forms a shallow, flat layer across the WOW dish may reinforce the developmental disadvantage of this condition in the present study. This likely contributes to suboptimal microenvironments within individual microwells due to inadequate medium depth and uneven coverage. In contrast, the 50 μL WOW condition may represent a better balance by providing sufficient medium volume to effectively dilute toxic metabolites, while still enabling the localized accumulation of beneficial autocrine and paracrine factors around each embryo. This dual advantage could explain the superior developmental outcomes observed in the 50 μL WOW group.

The direct comparison between the conventional porcine embryo culture (control) and 50 μL microwell (50 μL WOW) conditions again verified that for culturing porcine embryos, the microwell system is superior to the conventional drop system. Although there was no difference in the percentage of embryos cleaved, the percentage of blastocysts formed and the average number of cells in the blastocysts were higher in the microwell system, further confirming that culturing embryos in conventional drops, which is currently the preferred culture system for embryos, is not the most favorable for development [8]. When mouse embryos were incubated under various culture conditions, the microwell system did not improve blastocyst formation compared to the traditional culture drops but increased the average number of nuclei in the inner cell mass of the blastocysts [14]. Similarly, when the development of pig embryos generated by intracytoplasmic sperm injection (ICSI) was studied, it was found that the cleavage rate on day 2 and the mean cell number of the blastocysts on day 6 were similar among the different treatment groups, but the rate of blastocyst formation improved significantly in the microwells compared to the conventional culture drop system [13]. In our experiment, not only the blastocyst rate but the average cell number per blastocyst also improved significantly in the microwell system, which suggests that the factors postulated to promote blastocyst formation in the microwell system also support improvement in the quality of the embryos. While the averages of 33.21 ± 1.56 (control) and 38.97 ± 1.80 (50 μL WOW) nuclei per blastocyst in the present study may seem low, it aligns with reported total nuclei count outcome of porcine IVF blastocysts cultured under standard in vitro conditions [4]. The lower cell counts in some embryos also likely reflect inherent developmental variability and limitations of in vitro porcine embryo culture.

The unique characteristics of the microwell system may be responsible for its beneficial effects. It was proposed that the microwells create an environment that allows for the appropriate diffusion of the molecules produced by the embryos. As mentioned above, the embryos generate growth factors that promote their development and at the same time release waste products that are harmful to the embryos. Growth factors are known to be large proteins while waste materials such as NH_4_^+^, ROS, and TNF-α are small molecules with higher diffusion kinetics [34,35,36]. Using numerical simulations, Matsuura calculated the concentrations of secreted molecules surrounding the embryos in various culture systems [37]. He found that the microwell system supported better development because it facilitated the diffusion of the small waste molecules out of the microwell and away from the embryos, while at the same time allowing for the accumulation of large growth-promoting macromolecules around the embryos. In addition, the dimensions of the microwell also seem to be important. Small-size microwells (130 µm inner diameter and 150 µm depth) supported a significantly higher cleavage rate and blastocyst formation in bovine embryos produced by handmade cloning compared to large-size microwells (280 µm inner diameter and 250 µm depth) [38]. It is possible that the more restricted space in the smaller microwells created a microenvironment around the embryos that provided a higher concentration of favorable factors and mimicked more closely the in vivo conditions of early pregnancy. Data from an ongoing study in our laboratory reveal that blastocyst development is improved in 2-cell and 4-cell porcine IVF embryos that had been initially cultured in conventional dishes and later transferred into fresh Well-of-the-Well (WOW) dishes. These findings suggest that beneficial embryo-derived factors are produced very early in sufficient quantities to support development. Compared to embryos cultured exclusively in conventional dishes, the improved blastocyst rates observed in embryos relocated to WOW at the 2-cell and 4-cell stages further underscore the efficacy of the microwell culture systems in promoting porcine blastocyst production.

## 5. Conclusions

In summary, this study reveals that while the individual culture of in vitro-produced porcine embryos in microwells did not affect cleavage rates, it significantly improved blastocyst formation and nuclear counts compared to the conventional culture medium droplet system. The microwell system provides a practical and effective approach, ideal for supporting the development of healthy in vitro fertilization-derived porcine embryos while allowing for individual tracking of development during the culture period.

## Data Availability

The original contributions presented in this study are included in the article. Further inquiries can be directed to the corresponding author.
